# A Huge Capital Drop with Compression of Femoral Vessels Associated with Hip Osteoarthritis

**DOI:** 10.1155/2015/709608

**Published:** 2015-10-04

**Authors:** Tomoya Takasago, Tomohiro Goto, Takahiko Tsutsui, Kenji Kondo, Daisuke Hamada, Ichiro Tonogai, Keizo Wada, Koichi Sairyo

**Affiliations:** Department of Orthopedics, Institute of Biomedical Sciences, Tokushima University Graduated School, Tokushima 770-8503, Japan

## Abstract

A capital drop is a type of osteophyte at the inferomedial portion of the femoral head commonly observed in hip osteoarthritis (OA), secondary to developmental dysplasia. Capital drop itself is typically asymptomatic; however, symptoms can appear secondary to impinge against the acetabulum or to irritation of the surrounding tissues, such as nerves, vessels, and tendons. We present here a case of unilateral leg edema in a patient with hip OA, caused by a huge bone mass occurring at the inferomedial portion of the femoral head that compressed the femoral vessels. We diagnosed this bone mass as a capital drop secondary to hip OA after confirming that the mass occurred at least after the age of 63 years based on a previous X-ray. We performed early resection and total hip arthroplasty since the patient's hip pain was due to both advanced hip OA and compression of the femoral vessels; moreover, we aimed to prevent venous thrombosis secondary to vascular compression considering the advanced age and the potent risk of thrombosis in the patient. A large capital drop should be considered as a cause of vascular compression in cases of unilateral leg edema in OA patients.

## 1. Introduction

Capital drop is a type of osteophyte commonly observed at the inferomedial portion of the femoral head secondary to hip subluxation in osteoarthritis (OA) due to developmental dysplasia [1]. Such osteophytes are thought to arise from the bone-cartilage junction through endochondral ossification; these have mature trabecular bone at the core with a peripheral cartilage layer. By itself, it does not adversely affect the symptoms of hip OA.

Unilateral leg edema usually occurs following vascular or lymphatic dysfunction. Elderly patients with various comorbidities, such as hypertension, arteriosclerosis, hyperlipidemia, or diabetes mellitus, are considered to be at an especially high risk of deep venous thrombosis. Leg edema caused by mechanical compression of the vessels around the hip joint is relatively rare, and only a few cases of synovial cyst of the hip causing vascular obstruction have been reported [2, 3]. Here, we present a rare case of unilateral leg edema caused by a huge capital drop that compressed the femoral vessels in hip OA.

## 2. Case Report

**Figure 1:**
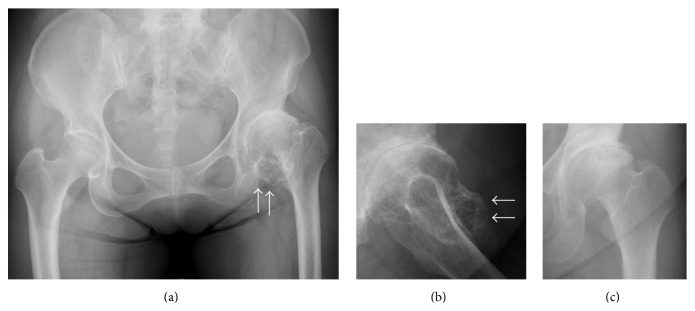
Radiographs of the left hip. Anteroposterior (a) and lateral (b) views of the left hip at initial presentation. The osseous mass (white arrows) appeared to have a connection to the femoral head on the mediolateral view of the X-ray. Anteroposterior X-ray obtained 8 years earlier (c). No abnormal bone mass is observed.

**Figure 2:**
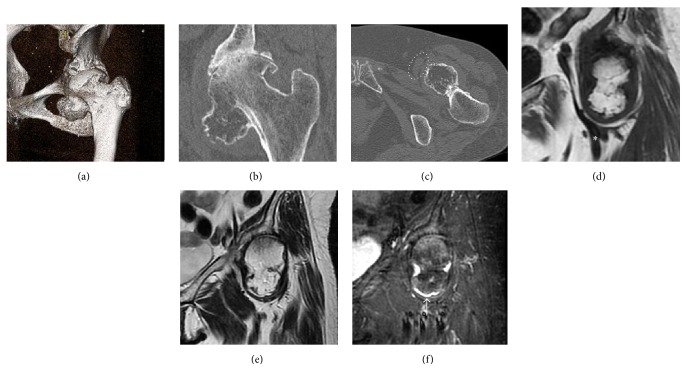
Computed tomography (CT) images and magnetic resonance imaging (MRI) of the left hip. Oblique coronal CT image of the left hip revealed a 40 × 40 × 30 mm sized bone mass connecting to the inferomedial portion of the femoral head. Continuity of the medullary bone is observed at the base of the mass (a, b). The bone mass compressing the femoral vessels is observed on CT (c) and MRI (d); white dotted circle and asterisk indicate the compressed femoral vessels. MRI revealed an intra-articular bone mass surrounded by joint effusion. The intensity of the mass is similar to that of the surrounding normal bone on both T1- (d) and T2-weighted images (e). The cartilage cap corresponding to the very high intensity area is observed at the periphery of the mass on the short-tau inversion recovery (STIR) image (white arrow) (f).

**Figure 3:**
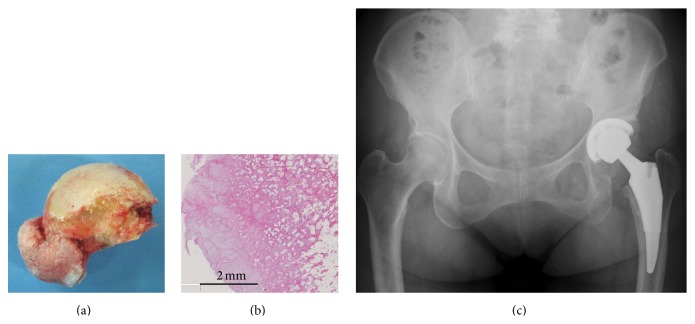
A photograph of the resected bone mass (a). Histological findings of the bone mass (b). Scale bar: 2 mm. Mature trabecular bone and a cartilage layer were noted on the surface. Postoperative radiographs of the hip showing the total femur prosthesis as implanted (c).

A 71-year-old Japanese woman presented with a 10-year history of left hip pain and 1-year history of left leg edema. The left leg edema was apparent particularly after long durations of standing, sitting, or walking. The patient had a history of hypertension, arteriosclerosis, and internal carotid artery constriction. We could feel pulses of the femoral and dorsal arteries well even when the leg edema appeared. Physical examination revealed limitation of the range of motion (ROM) of her left hip, with 80° flexion, 20° abduction, 15° adduction, 45° external rotation, and 5° internal rotation. Plain radiographs revealed advanced OA secondary to developmental dysplasia of the left hip. A huge osseous mass was also observed at the inferomedial portion of the femoral head (Figures 1(a) and 1(b)). Based on a previous radiograph, we determined that this mass was absent 8 years ago (Figure 1(c)). Computed tomography (CT) images and magnetic resonance imaging (MRI) revealed compression of the femoral vessels by the mass (Figures 2(a)–2(d)). On MRI, the intensity of the mass was almost similar to the surrounding bone marrow, with very high intensity at the surface on the short-tau inversion recovery (STIR) image (Figures 2(d)–2(f)). The Japanese orthopaedic association (JOA) hip score [4] was 54 points: 20 points in pain, 12 points in ROM, 10 points in gait, and 12 points in activity of daily life. We diagnosed the mass as a capital drop secondary to OA and performed total hip arthroplasty (THA) with resection of the mass. The mass was observed to be completely inside the articular capsule and there was minimal inflammation and adhesion between the mass and capsule. Cartilage tissue was observed on the surface of the mass (Figure 3(a)). THA was performed using a short tapered wedge stem with a 32 mm ceramic head (Microplasty, BIOMET, Warsaw, IN) and a 48 mm cementless cup with a polyethylene liner (Trident cup with X3 liner, Stryker, Mahwah, NJ) (Figure 3(b)). Histological findings revealed a peripheral cartilage layer, with mature trabecular bone present in the central zone. No malignant cells were observed (Figure 3(c)).

Following surgery, the left leg edema immediately diminished and the postoperative course was uneventful. At the latest follow-up, that is, 3 years after the surgery, the patient was able to walk without an aid and was independent in all activities of daily living, with no evidence of recurrence. The ROM of her left hip dramatically improved, with 120° flexion, 45° abduction, 20° adduction, 40° external rotation, and 20° internal rotation and the JOA hip score improved from 54 points preoperatively to 100 points at the latest follow-up.

## 3. Discussion

This case of an unusual, huge capital drop osteophyte secondary to hip OA was associated with compression of the femoral vessels, leading to leg edema. van der Kraan and van den Berg described that the osteophyte arises in the periosteum overlying the bone at the junction of the cartilage and bone through endochondral ossification, and they called the osteophyte “a fibrocartilage-capped bony outgrowth” [5].

Several reports of osteochondroma have described an appearance similar to that in our case, presenting as an osseous mass around the femoral neck [6–11]. Osteochondroma is the most common benign bone tumor, with most cases being a solitary lesion. Osteochondroma arises from the growth plate of the metaphysis of the long bone; thus, it typically exists around the joints of long bones. Most cases of osteochondroma show an increase in size during childhood but no growth after skeletal maturity [10]. Osteochondroma is typically asymptomatic; however, symptoms can appear secondary to compression or irritation of the surrounding tissues, such as nerves, vessels, and tendons [7, 9, 12, 13], which depend on the size and site of the lesion. Fractures [6, 11], impingement [8, 14], and snapping hip [10] may occur with an osteochondroma in the hip joint. In these patients, MRI shows an osseous prominence with low intensity at the periphery, corresponding to the cortical bone, and a central core of high intensity, corresponding to the bone marrow in T1- and T2-weighted images. Normal trabecular bone continuity between the original bone and the mass is also noted. Histologically, the tumor has hyaline cartilage in the peripheral zone (the so-called cartilage cap) and mature trabecular bone inside [11]. Based on imaging and histological findings, it is impossible to differentiate between osteophyte and osteochondroma, with both lesions showing mature trabecular bone inside the mass and a cartilage layer on the surface.

In the present case, the typical features of osteochondroma were noted on both imaging studies and histology; however, we diagnosed a capital drop osteophyte based on the patient's advanced age and a previous radiograph obtained 8 years ago, which revealed no osseous mass in the left hip. This indicated that the osseous mass had appeared at the age of 63 years or later. Since it was inconceivable that an osteochondroma could occur and grow in a patient aged over 60 years, we therefore considered it a capital drop osteophyte secondary to hip OA. To our knowledge, a case of a huge capital drop in hip OA that compressed the femoral vessels and resulted in unilateral leg edema has not been reported thus far. Although other treatment options, including conservative treatment or resection of the mass, may have been indicated for this case, we performed early resection and THA. This was because the cause of the patient's hip pain was not only the mass but also OA of the hip joint; moreover, we considered that the risk of venous thrombosis secondary to the vascular compression in this patient was high, given her advanced age and various comorbidities.

In summary, in cases of unilateral leg edema in OA patients, physicians should consider the possibility of a large capital drop causing vessel compression.
